# Another View of American Descendants of Slavery Representation in the American Global Health Community

**DOI:** 10.4269/ajtmh.21-0720a

**Published:** 2021-08-18

**Authors:** Frank Richards

**Affiliations:** Onchocerciasis, Lymphatic Filariasis, Schistosomiasis and Malaria Programs The Carter Center1 Copenhill, 453 Freedom Parkway Atlanta, GA 30307 E-mail: frank.richards@cartercenter.org

Dear Sir,

I enjoyed reading the timely Perspective Piece by Dr. Tishina Okegbe published in the May 2021 issue of the *American Society of Tropical Medicine and Hygiene*.^1^ I write to share some of my thoughts about it and to propose an additional recommendation. I draw from my perspectives as an American descendant of slavery (ADOS)^2^ and a physician who has been a member of American Society of Tropical Medicine and Hygiene (ASTMH) for over 35 years.

First, I struggle with being labeled an ADOS because it subliminally negates my family’s continued suffering past the 1865 Emancipation (happily declared a national holiday by President Joe Biden in June 2021). The ensuing 150+ years of social/financial/professional oppression heaped upon the descendants of slavery is commonly denied in the arguments I’ve heard since my coming of age in 1968: “Give me a break! Get over it! Slavery happened a long time ago and has nothing to do with me!”. How fitting that also in June 2021 we reminded ourselves of post–Civil War old and new Jim Crow^3^ terrorism by commemorating the 100th-year anniversary of the violent destruction of Tulsa’s “Black Wall Street.”

Second, I initially read Dr. Okegbe’s article with my “physician’s brain” turned on, and immediately stumbled on the use of the word “acutely” in the abstract (“ . . . they [ADOS] are acutely absent in this [global health] field,” which I interpreted as meaning an illnesses of short duration. Dr. Okegbe’s statement that ADOS were “acutely absent” from the American Global Health community literally led to my verbal outcry “ADOS underrepresentation is not an acute but a chronic illness!”

Third, additional historical highlights exist with respect to ADOS working on the global stage that ought to (together with Juneteenth and Tulsa) be remembered. One is the fascinating story of William Henry Sheppard, the son of American slaves, who stood against King Leopold of Belgium’s brutal colonialization of Congo.^4^ Leopold’s racist machine led to an estimated 10 million Congolese deaths (similar in magnitude to the 12–14 million estimated African deaths on the slave ships of the Middle Passage). At the risk of his life, Mr. Sheppard played a remarkable leadership role in the world’s first international human rights trial, which ultimately stripped Leopold’s (and some of Belgium’s) power over the “Congo Free State.”

Finally, I share Dr. Okegbe’s concerns about the lack of data on the racial and ethnic composition of the American global health workforce. I suspect she would agree with my proposing another recommendation: “Create data bases and monitoring dashboards/scorecards to establish the current (‘baseline’) racial and ethnic participation in global health, and then monitor indices of success of the new efforts to enhance inclusion over time.” We should now seek to reward solid results, not merely noble intentions that seem increasingly cliché. I am pleased that the leadership of many institutions, including that of the ASTMH, are examining not only their policies, but their monitoring and data systems. Unfortunately, as a child of the affirmative action generation, I sadly predict that this window of opportunity to take meaningful action for ADOS participation in global health will “acutely” close as global outrage wanes to the shock from viewing the videoed murders of Mr. George Floyd and others.
